# Recreational Cannabis Legalization and Traffic Fatality Rates in the United States, 2005-2024: An Imputation-Based Difference-in-Differences Event Study

**DOI:** 10.7759/cureus.109759

**Published:** 2026-05-27

**Authors:** Jose A Acosta, Israel Alvarado

**Affiliations:** 1 Basic Sciences, Ponce Health Sciences University, St. Louis, USA; 2 Medical Biochemistry and Microbiology, Ponce Health Sciences University, St. Louis, USA

**Keywords:** cannabis legalization, difference-in-differences, policy evaluation, recreational cannabis, road traffic fatalities

## Abstract

Background

Recreational cannabis legalization has expanded rapidly in the United States; however, population-level analyses examining its association with traffic fatalities yield mixed results. These heterogeneous findings arise from methodological limitations, including short follow-up windows, a focus on early-adopting states, and methods that do not adequately account for staggered policy adoption.

Methods

We analyzed a 50-state panel (2005-2024) using an imputation-based difference-in-differences estimator designed for staggered policy adoption. The outcome was the log traffic fatality rate per one billion vehicle miles traveled. We estimated effects from the year of legalization through year four, with four pre-treatment years to assess parallel trends. The primary model included state- and year-fixed effects and controlled for demographic composition, weather (precipitation and annual average temperature), unemployment, traffic safety laws, and COVID-19 mortality. Sensitivity analyses estimated robustness by varying the sample composition (excluding early adopters, pandemic years, and Florida), modifying the treatment definition (legalization effective date vs. retail sales start), and including Poisson count-model replication.

Results

The panel included 1,000 state-year observations; the primary difference-in-differences analysis used 951 observations meeting all inclusion criteria. Post-legalization, traffic fatality rates increased by approximately 4.2% in the legalization year (τ=0: 0.041; 95% confidence interval (CI): 0.006, 0.077; p=0.022) and 6.1% by year four (τ=4: 0.060; 95% CI: 0.007, 0.112; p=0.027). Pre-treatment trends were parallel between treatment and control groups (joint test: χ²(4)=4.93; p=0.295), and the post-legalization increase was statistically significant overall (joint test: χ²(5)=11.56; p=0.041). Findings remained consistent when excluding early-adopting states, pandemic years, or Florida, and a Poisson count-model replication produced the same direction and magnitude. When treatment was instead indexed to the start of retail sales, no increase was observed in the first year (τ = 0: 0.011; 95% CI: −0.027, 0.050), and the overall post-retail joint test did not reach conventional significance (*p* = 0.067).

Conclusion

Legalizing recreational cannabis was followed by a rise in traffic deaths during the first four years after the law took effect. The increase began at legalization rather than at the start of retail sales. This suggests that the change in law and shifting public attitudes had a greater impact on traffic safety than the availability of cannabis in stores. States considering legalization should prepare traffic safety measures in advance, while those that have already legalized should reinforce existing efforts to address ongoing risks.

## Introduction

Acute cannabis intoxication impairs cognitive and motor functions essential for driving. Experimental studies and meta-analyses demonstrate that intoxication degrades reaction time, lane-position control, and divided attention, increasing the risk of crash involvement [1-3]. Given that traffic fatalities remain a leading cause of injury death in the United States, claiming approximately 41,000 lives annually, this impairment risk carries substantial public health significance [4]. However, translating these laboratory findings to real-world crash outcomes has proven more complex.

In a large United States case-control study, alcohol was strongly associated with crash risk, whereas tetrahydrocannabinol (THC), the primary psychoactive constituent of cannabis, showed no statistically significant association with crash risk, underscoring the difficulty of linking cannabis intoxication to observed crashes [5]. Whether this complexity extends to population-level fatality trends following legalization remains an equally unsettled question. The stakes of that question have grown considerably as recreational cannabis laws have expanded in a staggered fashion, from Colorado and Washington in 2012 to 24 states by 2024 [6,7]. Early studies focusing on the first years after legalization reported no clear changes. In contrast, more recent work with longer follow-up has found increases in some settings and null or mixed effects in others [8-14].

These mixed findings likely reflect both true contextual differences and variation in follow-up duration, comparison groups, and analytic methods. A particular concern is that standard two-way fixed effects (TWFE) models, a common choice in prior work, can yield biased estimates under staggered policy adoption when early-adopting states are used as controls for later adopters [6,15,16]. As a result, the population-level impact of recreational legalization on traffic fatalities remains uncertain [14,17-19].

To address these methodological and design limitations, we aimed to determine whether recreational cannabis legalization affects traffic fatality rates relative to continued prohibition. We analyzed 20 years of United States national data (2005-2024) using an imputation-based difference-in-differences estimator that accommodates staggered policy adoption. We conducted sensitivity analyses that varied sample composition, compared alternative treatment definitions, and replicated results using a Poisson count model.

## Materials and methods

This was an observational, quasi-experimental policy evaluation study that used a longitudinal panel of United States states to estimate changes in traffic fatality rates following recreational cannabis legalization. The study period spanned 2005-2024, providing a 20-year panel with complete data for key covariates. The study was approved (exempt) by the Ponce Research Institute Institutional Review Board (protocol number 2601301151, document number: 183292), and adhered to the Strengthening the Reporting of Observational Studies in Epidemiology (STROBE) guidelines [20], with additional consideration of reporting practices specific to difference-in-differences designs (see Appendix A). The primary outcome of the study was the log-transformed traffic fatality rate per one billion vehicle miles traveled (VMT). 

Data sources

Fatality counts were derived from the National Highway Traffic Safety Administration (NHTSA) Fatality Analysis Reporting System (FARS) [21] and combined with traffic exposure data from the Federal Highway Administration (FHWA) [22]. A natural log transformation was applied to enable interpretation of coefficients as approximate percentage changes. Fatality counts for 2024 were provisional at the time of analysis [23]. These fatality totals serve as the baseline for assessing our primary exposure: the implementation of recreational cannabis laws.

Exposure definition

The primary exposure for this analysis was recreational cannabis legalization. For the purpose of this study, this exposure is defined as the first calendar year in which a state’s adult nonmedical cannabis law was officially in effect. This calendar-year coding captures the policy-relevant change in legal status. It aligns with the annual aggregation of the outcome, recognizing that implementation dates within a given year may vary across states. Using statutory effective dates and policy surveillance resources, we independently identified the timing of this legal status change, separate from the subsequent initiation of retail sales [24,25]. These dates allowed us to create a treatment-timing variable equal to the year of legalization, while states that maintained prohibition throughout the study period were coded as never-treated. The complete state-by-state timeline of recreational and medical cannabis legalization used in the analytic panel is provided in Appendix B.

Covariates

We adjusted for time-varying state characteristics related to four domains: demographic composition, driving environment, economic conditions, and COVID-19 severity. To capture demographic composition, we included the annual percentage of the population that was pediatric (0-17 years), male, and White, as well as the percentage living in rural areas. Rural classification was constructed from county-level rural-urban classifications and population counts [26]. Population data came from the National Cancer Institute, Surveillance, Epidemiology, and End Results (SEER) program (2005-2023) [27] and the United States Census Population Estimates Program (PEP) (2024) [28].

To capture the driving environment, we adjusted for annual state-level precipitation, annual average state temperature, the presence of a primary seat belt enforcement law, and the maximum posted speed limit on rural Interstate highways, using a reconstructed state-year speed limit panel [29,30]. Economic conditions were represented by the annual state unemployment rate from the Bureau of Labor Statistics [31]. Finally, to account for differences in pandemic severity beyond year fixed effects, we included a measure of COVID-19 mortality for 2020 and 2021, expressed as deaths per 100,000 population; values were set to zero for pre-pandemic years [32]. Full variable definitions, coding decisions, and data sources are provided in Appendix C.

Statistical analysis

To address potential bias from staggered policy adoption, we estimated dynamic effects using the imputation-based difference-in-differences estimator of Borusyak et al. [33]. This approach fits an outcome model using untreated state-year observations, never-treated states, and pre-treatment periods to predict counterfactual outcomes. In other words, the model predicts what would have happened in legalizing states had they remained under prohibition, then computes the average treatment effect on the treated (ATT) by comparing these predictions to observed fatality rates.

In our primary specification, we estimated effects over five event times, from the legalization year through year four (t = 0 to t = 4). We also included four placebo lead coefficients (t = −4 to t = −1) to assess the parallel-trends and no-anticipation assumptions. We restricted the estimation to a four-year post-treatment horizon to ensure that a small subset of early-adopting states does not disproportionately drive long-run estimates. Because event-time effects were estimated only within four pre- and five post-treatment periods, observations outside this window did not affect those estimates but were kept to estimate fixed effects. Standard errors were clustered at the state level to account for within-state serial correlation, and inference used two-sided tests with 95% confidence intervals (CI).

Sensitivity analyses addressed potential concerns about sample composition, treatment definition, and model specification. To evaluate sample composition, we re-estimated the model after excluding the two earliest-adopting states (Colorado and Washington), the pandemic years (2020 and 2021), and the state with the largest population denominator discrepancy (Florida). To evaluate treatment definition, we indexed event time to the start of retail sales rather than the statutory legalization date, and we restricted the sample to states with observed retail operations while retaining legalization-date indexing as an apples-to-apples within-sample comparison. We also re-estimated the model using the effective year of legalization rather than the passage year as the treatment-coding variable. Finally, we replicated the primary analysis using a Poisson regression model with state- and year-fixed effects, total fatality counts as the outcome, VMT as an exposure offset, and standard errors clustered by state.

Because the number of treated states is fixed by policy adoption, we did not conduct formal power calculations. The final estimation sample included 24 treated states and 26 never-treated controls observed over 20 years (2005-2024), yielding 951 state-year observations for the primary specification. 

All statistical procedures were performed using Stata Statistical Software: Release 19 (StataCorp LLC, College Station, Texas, United States). We report two-sided p-values and 95% CIs, with p < 0.05 as the conventional significance threshold.

Identifying assumptions

The validity of our estimates depends on several identifying assumptions [33]. The parallel trends assumption requires that fatality trends in treated and control states would have evolved similarly absent legalization; we assessed this by examining pre-treatment coefficients and conducting a joint test of leads. The no-anticipation assumption requires that outcomes remain unchanged until the legal effective date. We addressed potential composition bias by restricting the post-treatment horizon to four years, preventing estimates from being disproportionately driven by early-adopting states. The stable unit treatment value assumption (SUTVA) requires that legalization in one state does not directly affect fatality rates in another; we acknowledge this as an identifying assumption that may be imperfect if cross-border effects exist. Finally, consistency assumes that the treatment (recreational cannabis legalization) is sufficiently well-defined across jurisdictions.

## Results

The state-year panel contained 1,000 observations, representing 50 United States states across 20 years. Of these, 951 observations contributed to the specific event-time estimates within the primary estimation window, spanning four pre-treatment and five post-treatment periods (τ = −4 through τ = 4). The remaining 49 observations represented periods of more than four years post-legalization in early-adopting states. These observations were retained in the model to support the estimation of fixed effects but were not assigned a discrete-event-time coefficient.

Twenty-four states legalized recreational cannabis during the study period. Table 1 shows baseline characteristics by legalization status. Compared with never-legalizing states, legalizing states were less rural, had lower baseline fatality rates, were cooler on average, and had higher pre-legalization unemployment.

**Table 1:** Descriptive statistics for the state-year panel, United States (2005–2024) Values represent mean (standard deviation) for continuous variables and are untransformed rates. Legalization refers to recreational cannabis legalization. 1B VMT: one billion vehicle miles traveled.

Variable	Overall (N=1000)	Never legalized (n=520)	Pre-legalization (n=330)	Post-legalization (t=0…4) (n=101)	Post-legalization (t≥5) (n=49)
Fatality rate per 1B VMT	12.4 (3.3)	13.6 (3.1)	10.9 (3.2)	11.3 (3.0)	11.9 (2.7)
% Pediatric population (0–17)	23.1 (2.1)	23.7 (2.2)	23.0 (1.6)	21.5 (1.6)	21.1 (1.8)
% Male population	49.5 (0.8)	49.6 (0.7)	49.3 (0.8)	49.7 (0.8)	50.3 (1.0)
% White population	80.8 (12.6)	80.3 (15.3)	81.5 (8.5)	80.4 (8.9)	81.2 (8.6)
% Rural population	24.5 (17.5)	30.5 (15.2)	18.2 (18.1)	17.6 (16.9)	17.1 (14.9)
Annual precipitation (inches)	38.6 (15.4)	40.8 (16.0)	37.3 (14.5)	34.7 (14.2)	33.0 (13.6)
Annual average temperature (°F)	53.0 (8.6)	55.8 (9.0)	50.4 (6.9)	50.2 (7.2)	46.7 (7.9)
Primary seat belt law (% yes)	63.1 (48.3)	66.2 (47.4)	58.8 (49.3)	61.4 (48.9)	63.3 (48.7)
Unemployment rate (%)	5.4 (2.2)	5.0 (2.1)	6.2 (2.2)	4.7 (2.0)	4.6 (1.5)
Max rural Interstate speed limit (mph)	70.9 (5.0)	72.3 (4.7)	68.6 (4.8)	70.6 (4.9)	71.8 (4.8)

**Figure 1:**
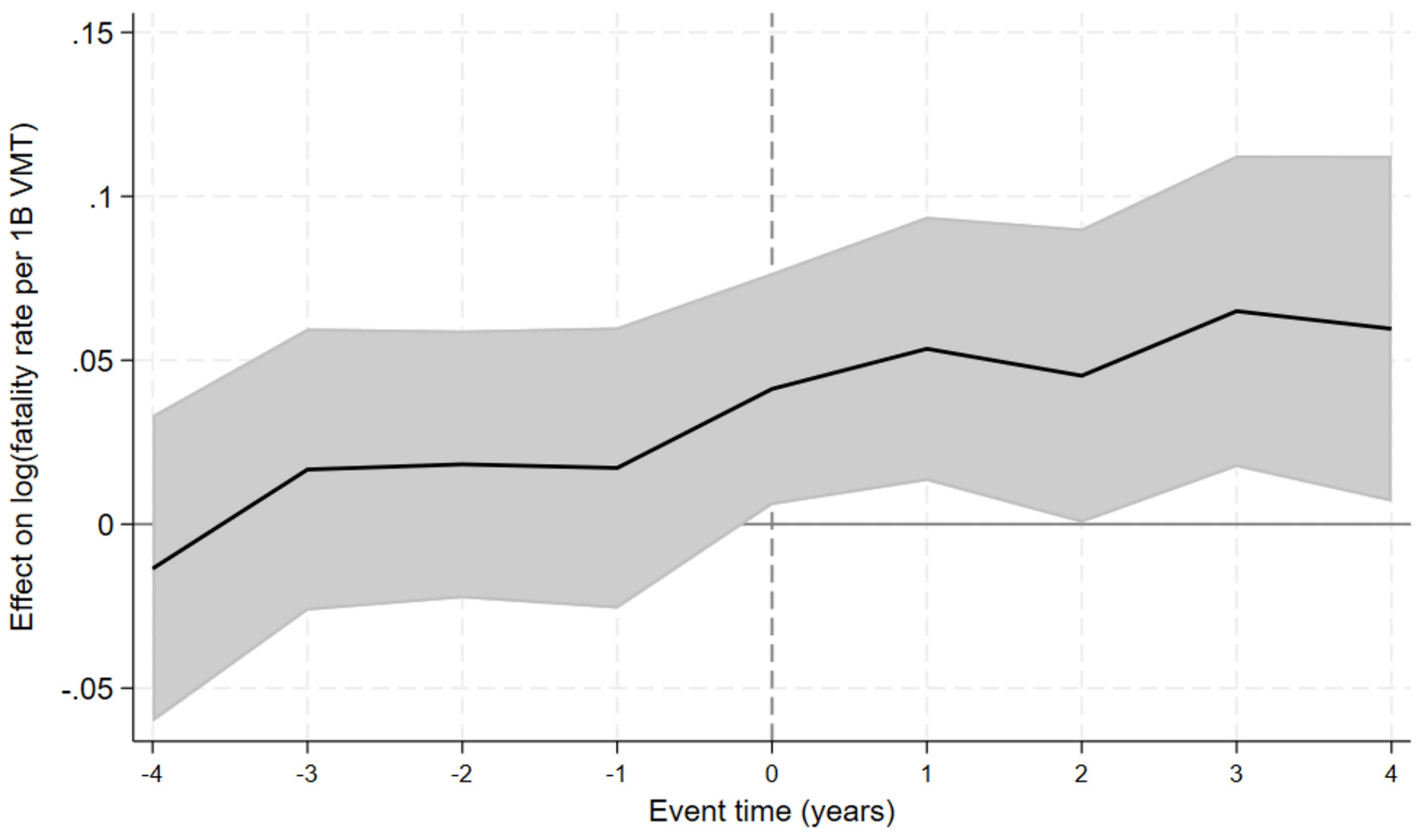
Event-study estimates for recreational cannabis legalization and log traffic fatality rate per 1B VMT DID-imputation event-study coefficients with 95% CIs from the primary specification. The outcome is the log traffic fatality rate per 1B VMT. Coefficients can be interpreted as approximate percent changes in the fatality rate. Event time t is defined relative to the legalization year (t = 0); negative values are years before legalization, and positive values are years after. Vertical dashed line separates the pre-period from the legalization year (t=0). 1B VMT: 1 billion vehicle miles traveled; DID: difference-in-differences

Figure 1 and Table 2 present the primary event-study estimates. States that legalized recreational cannabis experienced estimated increases in traffic fatality rates of approximately 4.2% in the legalization year (τ=0: 0.041; 95% CI: 0.006, 0.077; p=0.022) and 6.1% by year four (τ=4: 0.060; 95% CI: 0.007, 0.112; p=0.027). Pre-treatment trends were parallel between groups (joint test: χ²(4)=4.93; p=0.295), consistent with the parallel trends assumption. The overall post-legalization increase was statistically significant (joint test: χ²(5)=11.56; p=0.041).

**Table 2:** Imputation-based difference-in-differences event-study estimates for log traffic fatality rate per 1B VMT Estimates are ATT at event time τ relative to the year of recreational cannabis legalization (τ=0). The outcome is the natural log of the fatality rate per 1 billion VMT. The model includes state and year fixed effects and adjusts for time-varying covariates (percent rural, demographic composition, precipitation, annual average temperature, primary seat belt law, unemployment rate, maximum rural Interstate speed limit, and COVID-19 mortality severity controls for 2020 and 2021). Percentage change is calculated as (exp(coefficient) − 1) × 100. Standard errors clustered by state. 1B VMT: 1 billion vehicle miles traveled; ATT: average treatment effects on the treated

Event time (years)	Coefficient	SE	95% CI	p-value	%Change
−4	−0.014	0.024	−0.060, 0.033	0.570	−1.4%
−3	0.017	0.022	−0.026, 0.060	0.447	+1.7%
−2	0.018	0.021	−0.023, 0.059	0.381	+1.8%
−1	0.017	0.022	−0.026, 0.060	0.433	+1.7%
0 (legalization year)	0.041	0.018	0.006, 0.077	0.022	+4.2%
1	0.053	0.021	0.013, 0.094	0.009	+5.5%
2	0.045	0.023	0.000, 0.090	0.048	+4.6%
3	0.065	0.024	0.017, 0.112	0.007	+6.7%
4	0.060	0.027	0.007, 0.112	0.027	+6.1%
Joint pre-trend test			χ²(4)=4.93	0.295	
Joint post-treatment test			χ²(5)=11.56	0.041	
State-years (N)	951				

Sensitivity analyses

Sensitivity analyses supported the robustness of the primary findings. Excluding the two earliest-adopting states (Colorado and Washington), excluding pandemic years (2020-2021), or excluding Florida produced similar event-time patterns and statistically significant post-treatment effects (Table 3). Population denominator comparisons between the SEER and Census PEP datasets showed minimal differences across the 50 states (Appendix D), with Florida identified as the only state with a discrepancy exceeding 1% (Appendix E). Excluding Florida did not materially affect the interim versus post-retail contrast (Appendix F) or the dynamic event-study estimates under either legalization timing (Appendix G) or retail timing (Appendix H).

**Table 3:** Sensitivity analyses: imputation-based difference-in-differences event-study estimates for log traffic fatality rate per 1 billion vehicle miles traveled Values are event-study coefficients with 95% CIs in parentheses. Event time 0 is the year of recreational cannabis legalization (columns (1)–(3)) or the year retail sales began (column (4)); negative values are pre-treatment years. All models include state and year fixed effects, time-varying covariates (percent rural, demographic composition, precipitation, annual average temperature, primary seat belt law, unemployment rate, maximum rural Interstate speed limit), and COVID-19 severity controls for 2020 and 2021. Standard errors clustered by state. * p < 0.05; ** p < 0.01

Event time (years)	(1) Primary specification	(2) Excluding CO & WA	(3) Excluding 2020–2021	(4) Retail sales timing
−4	−0.014 (−0.061, 0.033)	−0.021 (−0.070, 0.029)	−0.018 (−0.065, 0.029)	0.024 (−0.014, 0.062)
−3	0.017 (−0.026, 0.060)	0.017 (−0.029, 0.064)	0.011 (−0.036, 0.058)	−0.010 (−0.058, 0.037)
−2	0.018 (−0.023, 0.059)	0.022 (−0.024, 0.068)	−0.004 (−0.046, 0.038)	0.040* (0.001, 0.078)
−1	0.017 (−0.026, 0.060)	0.015 (−0.029, 0.060)	−0.004 (−0.057, 0.050)	0.045 (−0.004, 0.094)
0 (policy year)	0.041* (0.006, 0.077)	0.050* (0.013, 0.087)	0.025 (−0.023, 0.073)	0.011 (−0.027, 0.050)
1	0.053** (0.013, 0.094)	0.061* (0.019, 0.103)	0.059* (0.013, 0.104)	0.037 (−0.001, 0.076)
2	0.045* (0.000, 0.090)	0.052* (0.004, 0.100)	0.030 (−0.021, 0.081)	0.015 (−0.025, 0.055)
3	0.065** (0.017, 0.112)	0.061* (0.009, 0.113)	0.057* (0.008, 0.106)	0.044 (−0.023, 0.110)
4	0.060* (0.007, 0.112)	0.063* (0.007, 0.119)	0.067* (0.013, 0.121)	0.047* (0.008, 0.087)
Joint pre-trend test	χ²(4)=4.93; p=0.295	χ²(4)=6.55; p=0.162	χ²(4)=1.93; p=0.748	χ²(4)=5.99; p=0.200
Joint post-treatment test	χ²(5)=11.56; p=0.041	χ²(5)=15.77; p=0.008	χ²(5)=13.38; p=0.020	χ²(5)=10.31; p=0.067
State-years (N)	951	927	863	971

When treatment timing was indexed to the start of retail sales rather than legalization, effects emerged later and were attenuated. The initial-year coefficient was close to zero (τ = 0: 0.011; 95% CI: −0.027, 0.050), and the overall post-treatment joint test did not reach conventional significance (χ²(5) = 10.31; p = 0.067; Table 3, column 4). Only the four-year coefficient reached significance (τ = 4: 0.047; 95% CI: 0.008, 0.087; p = 0.018).

An additional sensitivity analysis that indexed treatment timing to the effective year of legalization (rather than the passage year) yielded broadly similar directional results. However, some estimates were attenuated and no longer statistically significant (Appendix I). A Poisson count-model specification produced estimates of similar direction and magnitude (Appendix J).

## Discussion

In this 20-year national panel of United States states, recreational cannabis legalization was associated with increases in traffic fatality rates during the first four years after adoption. Estimated increases ranged from approximately 4.2% in the legalization year to 6.1% by year four, with post-treatment estimates consistently positive and statistically significant in the primary specification.

Several design features strengthen confidence in these estimates. First, the analysis draws on a 20-year national panel that is substantially longer than the follow-up windows used in earlier evaluations, allowing both short- and medium-term effects to be characterized. Second, we used an imputation-based difference-in-differences estimator designed for staggered policy adoption [33], which avoids the bias that standard two-way fixed-effects models can introduce when early adopting states serve as controls for later adopters. Third, we tested the parallel-trends and no-anticipation assumptions through pre-treatment leads and a joint test and examined robustness through multiple sensitivity analyses (excluding early adopters, pandemic years, and Florida; alternative treatment-coding choices; and a Poisson count-model replication). Finally, we distinguished between the statutory legalization date and the onset of retail sales, a distinction that varies in how it is handled across the literature.

Our estimated post-legalization increases align with the broader literature, which shows that estimates vary by geographic scope, follow-up duration, and the operational definition of legalization [34,35]. A recent national analysis reported an approximately 4% increase in traffic fatality rates following legalization, a magnitude similar to our estimates [36]. Other studies report larger increases when allowing effects to vary across states and over time, while some find mixed or null associations [6,7]. Methodological differences contribute to this variation. For example, an interrupted time series analysis reported a transient increase in traffic fatalities following the onset of recreational retail sales, followed by a declining trend [14]. Together, these findings highlight the sensitivity of estimated effects to how legalization is defined and reinforce the importance of examining the timing of changes relative to specific policy milestones.

A key contribution of this study is clarifying the timing of changes in traffic fatality rates relative to policy milestones under a difference-in-differences framework. Because the onset of retail sales often lags statutory legalization, prior evaluations have frequently defined treatment as the start of commercial sales, a choice that can shift the apparent timing of effects [6,13,14]. By distinguishing between legalization and commercialization, this analysis highlights how alternative definitions of treatment can influence estimated associations. The findings suggest that observed changes in fatality rates are more closely aligned with the change in legal status than with the onset of retail operations.

Limitations

Several limitations should be noted. First, we cannot attribute observed increases to cannabis-impaired driving specifically, as our outcome measures total traffic fatality rates. Nonetheless, these population-level effects remain central for policymakers assessing road safety risks of legalization. Second, recreational cannabis legalization encompasses multiple policy components, including retail timing, product potency, taxation, and local opt-out provisions, and the individual contribution of each component cannot be isolated in this analysis. Third, using annual state-level data means that changes within a given year cannot be examined, potentially obscuring short-term changes in traffic fatality rates. Residual confounding remains possible despite fixed effects and covariate adjustment, as unmeasured time-varying factors may correlate with both legalization timing and fatality trends. Event-study designs have limited power to detect pre-trend differences; although joint pre-trend tests support the parallel trends assumption, they cannot definitively confirm it. Finally, our findings may not generalize to settings beyond the United States.

## Conclusions

Recreational cannabis legalization was followed by increases in traffic fatality rates during the first four years post adoption. Estimated increases began at legalization rather than at the onset of retail sales, consistent with the interpretation that legal status changes and shifting social norms may influence traffic safety more than commercial market availability. Retail-timing analyses produced weaker and later effects, but the retail-timing “pre-treatment” periods are mechanically contaminated by post-legalization observations and therefore cannot be interpreted as a clean causal counterfactual.

These findings highlight the importance of integrating traffic safety into cannabis policy reform. States should pair legalization with robust surveillance systems, targeted public education, and enhanced impaired-driving enforcement to mitigate these public health risks. By demonstrating that increases emerge at legalization rather than retail opening, and by using methods that account for staggered adoption across two decades, this study may help clarify prior methodological ambiguities and provide a firmer empirical foundation for evidence-based cannabis policy reform.

## Appendices

Appendix A

**Table 4:** STROBE statement: checklist of items that should be included in reports of cross-sectional studies STROBE: Strengthening the Reporting of Observational Studies in Epidemiology

	Item No.	Recommendation	Page(s)
Title and abstract	1	(a) Indicate the study’s design with a commonly used term in the title or the abstract	1
		(b) Provide in the abstract an informative and balanced summary of what was done and what was found	1–3
Introduction			
Background/rationale	2	Explain the scientific background and rationale for the investigation being reported	3–4
Objectives	3	State specific objectives, including any prespecified hypotheses	4
Methods			
Study design	4	Present key elements of study design early in the paper	5
Setting	5	Describe the setting, locations, and relevant dates, including periods of recruitment, exposure, follow-up, and data collection	5–6
Participants	6	(a) Give the eligibility criteria, and the sources and methods of selection of participants	5, 10
Variables	7	Clearly define all outcomes, exposures, predictors, potential confounders, and effect modifiers. Give diagnostic criteria, if applicable	5–7
Data sources/ measurement	8*	For each variable of interest, give sources of data and details of methods of assessment (measurement). Describe comparability of assessment methods if there is more than one group	5–7; Appendix A
Bias	9	Describe any efforts to address potential sources of bias	9–10
Study size	10	Explain how the study size was arrived at	9
Quantitative variables	11	Explain how quantitative variables were handled in the analyses. If applicable, describe which groupings were chosen and why	5–6
Statistical methods	12	(a) Describe all statistical methods, including those used to control for confounding	8–10
		(b) Describe any methods used to examine subgroups and interactions	N/A
		(c) Explain how missing data were addressed	8–10
		(d) If applicable, describe analytical methods taking account of sampling strategy	N/A
		(e) Describe any sensitivity analyses	9, 13–15
Results			
Participants	13*	(a) Report numbers of individuals at each stage of study—e.g., numbers potentially eligible, examined for eligibility, confirmed eligible, included in the study, completing follow-up, and analysed	10
		(b) Give reasons for non-participation at each stage	10
		(c) Consider use of a flow diagram	N/A
Descriptive data	14*	(a) Give characteristics of study participants (e.g., demographic, clinical, social) and information on exposures and potential confounders	11–12, Table 1
		(b) Indicate number of participants with missing data for each variable of interest	10
Outcome data	15*	Report numbers of outcome events or summary measures	12–13
Main results	16	(a) Give unadjusted estimates and, if applicable, confounder-adjusted estimates and their precision (e.g., 95% confidence interval). Make clear which confounders were adjusted for and why they were included	12–13
		(b) Report category boundaries when continuous variables were categorized	N/A
		(c) If relevant, consider translating estimates of relative risk into absolute risk for a meaningful time period	17
Other analyses	17	Report other analyses done—e.g., analyses of subgroups and interactions, and sensitivity analyses	13–14
Discussion			
Key results	18	Summarise key results with reference to study objectives	15–16
Limitations	19	Discuss limitations of the study, taking into account sources of potential bias or imprecision. Discuss both direction and magnitude of any potential bias	17–18
Interpretation	20	Give a cautious overall interpretation of results considering objectives, limitations, multiplicity of analyses, results from similar studies, and other relevant evidence	15–19
Generalisability	21	Discuss the generalisability (external validity) of the study results	18
Other information			
Funding	22	Give the source of funding and the role of the funders for the present study and, if applicable, for the original study on which the present article is based	N/A

Appendix B

**Table 5:** State-by-state recreational and medical cannabis legalization timeline of all 50 states of the United States (as of December 31, 2024) Passage year is the calendar year in which the recreational cannabis law was enacted by the legislature or approved by ballot initiative. Effective year is the calendar year in which the statute came into legal effect (used for the primary specification’s treatment-timing variable). Retail year is the calendar year in which licensed recreational retail sales began (used for the retail-timing sensitivity analysis; column (4) of Table 3). Medical cannabis laws are documented for descriptive purposes but were not used as an exposure in the primary or sensitivity analyses. Em dashes (—) denote not applicable. The analytic panel comprises 24 recreational-legalizing states (treated) and 26 never-recreational states (controls); medical-only states serve as never-treated controls for the recreational exposure. Sources: Compiled from Public Health Law Research, Recreational Marijuana Laws [24]; and the National Conference of State Legislatures, State Cannabis Policy Enactment Database [25]. Dates were cross-checked against state statutes and ballot-initiative records as needed.

State	Recreational law	Passage year	Effective year	Retail year	Medical law	Medical passage year
Alaska	Yes	2014	2015	2016	Yes	1998
Alabama	No	—	—	—	Yes	2021
Arkansas	No	—	—	—	Yes	2016
Arizona	Yes	2020	2020	2021	Yes	2010
California	Yes	2016	2016	2018	Yes	1996
Colorado	Yes	2012	2012	2014	Yes	2000
Connecticut	Yes	2021	2021	2023	Yes	2012
Delaware	Yes	2023	2023	—	Yes	2011
Florida	No	—	—	—	Yes	2016
Georgia	No	—	—	—	Yes	2015
Hawaii	No	—	—	—	Yes	2000
Iowa	No	—	—	—	Yes	2017
Idaho	No	—	—	—	No	—
Illinois	Yes	2019	2020	2020	Yes	2013
Indiana	No	—	—	—	No	—
Kansas	No	—	—	—	No	—
Kentucky	No	—	—	—	Yes	2023
Louisiana	No	—	—	—	Yes	2015
Massachusetts	Yes	2016	2016	2018	Yes	2012
Maryland	Yes	2022	2023	2023	Yes	2013
Maine	Yes	2016	2017	2020	Yes	1999
Michigan	Yes	2018	2018	2019	Yes	2008
Minnesota	Yes	2023	2023	2025	Yes	2014
Missouri	Yes	2022	2022	2023	Yes	2018
Mississippi	No	—	—	—	Yes	2022
Montana	Yes	2020	2021	2022	Yes	2004
North Carolina	No	—	—	—	No	—
North Dakota	No	—	—	—	Yes	2016
Nebraska	No	—	—	—	Yes	2024
New Hampshire	No	—	—	—	Yes	2013
New Jersey	Yes	2020	2021	2022	Yes	2010
New Mexico	Yes	2021	2021	2022	Yes	2007
Nevada	Yes	2016	2017	2017	Yes	1998
New York	Yes	2021	2021	2022	Yes	2014
Ohio	Yes	2023	2023	2024	Yes	2016
Oklahoma	No	—	—	—	Yes	2018
Oregon	Yes	2014	2014	2015	Yes	1998
Pennsylvania	No	—	—	—	Yes	2016
Rhode Island	Yes	2022	2022	2022	Yes	2006
South Carolina	No	—	—	—	No	—
South Dakota	No	—	—	—	Yes	2020
Tennessee	No	—	—	—	No	—
Texas	No	—	—	—	Yes	2015
Utah	No	—	—	—	Yes	2018
Virginia	Yes	2021	2021	—	Yes	2020
Vermont	Yes	2020	2020	2022	Yes	2004
Washington	Yes	2012	2012	2014	Yes	1998
Wisconsin	No	—	—	—	No	—
West Virginia	No	—	—	—	Yes	2017
Wyoming	No	—	—	—	No	—

Appendix C

Construction and Sources of Time-Varying Covariates, 2005-2024

1. Overview: This appendix documents the construction and data sources for time-varying covariates included in the main analysis. The covariates capture four domains: demographic composition, driving environment, economic conditions, and COVID-19 severity.

2. Demographic composition: We obtained annual state-level population counts by sex, age group, and race from the National Cancer Institute's Surveillance, Epidemiology, and End Results (SEER) Program for 2005-2023 [27] and the U.S. Census Bureau Population Estimates Program (Vintage 2024) for 2024 [28]. We constructed: (i) the percent pediatric population (ages 0-17), (ii) the percent male population, and (iii) the percent White population, each defined as the proportion of the state's total resident population. To assess consistency between SEER and Census denominators in 2023, we calculated percentage differences and flagged states with discrepancies >1%.

3. Rural population share: We constructed a time-varying measure of the percent rural population using the U.S. Department of Agriculture's Rural-Urban Continuum Codes [26] combined with annual population counts. For each state-year, we calculated the share of residents living in counties classified as non-metropolitan, capturing shifts in the urban-rural mix over time.

4. Driving environment: Maximum rural Interstate speed limit (mph): We constructed a state-year panel capturing statutory maximum posted speed limits for passenger vehicles on rural Interstate highways. The 2024 baseline values came from the Insurance Institute for Highway Safety (IIHS) database [30]. Historical changes (2005-2024) were compiled through a systematic review of state legislative records and Department of Transportation announcements. Each change was verified against at least two independent sources. The variable represents the maximum limit applying broadly across a state's rural Interstate system. For Texas, we coded the maximum as 80 mph (the Interstate limit) rather than 85 mph (which applies only to State Highway 130, a toll road). For states with multiple documented changes, we retained only the earliest change to maintain a clean staggered adoption design for difference-in-differences analysis.

Annual precipitation: We used state-level annual total precipitation (inches) from the National Oceanic and Atmospheric Administration's climate divisional database [29], aggregating monthly data to calendar-year totals.

Primary seat belt law: We coded a binary indicator for whether a state had primary seat belt enforcement in each year, using IIHS compilations and historical law summaries [30].

5. Economic conditions: We obtained annual state-level unemployment rates from the U.S. Bureau of Labor Statistics Local Area Unemployment Statistics (LAUS) program [31].

6. COVID-19 severity: To account for differential COVID-19 impacts beyond year fixed effects, we constructed a measure of COVID-19 mortality severity for 2020-2021 using state-level death counts from the National Center for Health Statistics [32]. We expressed this as deaths per 100,000 population and included it only for 2020 and 2021; values were set to zero for pre-pandemic years.

7. Annual average temperature (°F): We used state-level annual average temperature from the National Oceanic and Atmospheric Administration (NOAA) National Centers for Environmental Information climate database [29]. Monthly data were aggregated to calendar-year averages for each state.

Appendix D

**Table 6:** SEER vs. Census Vintage 2024 population overlap (2023; 50 states). Note. Summary statistics of percent differences between SEER program county population denominators (aggregated to state) and U.S. Census Bureau PEP Vintage 2024 revised estimates for 2023, across all 50 states. Percent difference is defined as 100 × (Census − SEER) / SEER. Values indicate close agreement between the two denominator sources, with 49 of 50 states within ±1%. PEP: Population Estimates Program; SEER: Surveillance, Epidemiology, and End Results

Metric (Diff = 100 × (Census − SEER) / SEER)	Value
Mean Diff (%)	0.42
Median Diff (%)	0.39
25th percentile Diff (%)	0.26
75th percentile Diff (%)	0.60
Min Diff (%)	−0.13
Max Diff (%)	1.30
States with |Diff| < 0.1%	3
States with 0.1% ≤ |Diff| ≤ 1%	46
States with |Diff| > 1%	1

 Appendix E

**Table 7:** Detail for outlier state with SEER vs. Census Vintage 2024 discrepancy exceeding 1% (2023) Note. Florida was the only state among the 50 with an absolute percent difference greater than 1% between SEER and Census Vintage 2024 population denominators in 2023. Percent difference is defined as 100 × (Census − SEER) / SEER. SEER: Surveillance, Epidemiology, and End Results.

State	SEER 2023	Census 2023	Diff (%)
Florida	22,610,726	22,904,868	1.30

Appendix F

**Table 8:** Sensitivity analysis excluding Florida — interim vs. post-retail comparison within the legalization-timing event study (R = 1). Contrast tests whether the mean of interim (pre-retail) post-legalization effects (τ₀–τ₂) differs from the mean of post-retail effects (τ₃–τ₄), where R = 2 is the median legalization-to-retail lag across the 19 states with both timings observed within the study window. Estimates from the Borusyak et al. [33] imputation-based DID estimator (did_imputation) with state and year fixed effects, time-varying covariates as in the primary specification, and standard errors clustered by state. Coefficients are in log points of the traffic fatality rate per 1 billion VMT. The legalization-timing event study was estimated on N = 951 state-years (full sample) and N = 931 (excluding Florida). DID: difference-in-differences; SE, standard error; VMT: vehicle miles traveled

Sample	M(τ₀–τ₁) − M(τ₂–τ₄)	SE	p	95% CI
Full sample	−0.009	0.013	.476	[−0.035, 0.016]
Excluding Florida	−0.009	0.013	.508	[−0.034, 0.017]

Appendix G

**Table 9:** Sensitivity analysis excluding Florida — dynamic treatment effects (τ₀–τ₄), legalization timing Cell entries are event-time coefficients with standard errors in parentheses. Event time 0 is the year of recreational cannabis legalization. Estimates from the Borusyak et al. (2024) [33] imputation-based DID estimator (did_imputation) with state and year fixed effects, time-varying covariates as in the primary specification (percent rural, demographic composition, precipitation, annual average temperature, primary seat belt law, unemployment rate, maximum rural Interstate speed limit, and COVID-19 severity controls for 2020 and 2021), and standard errors clustered by state. Coefficients are in log points of the traffic fatality rate per 1 billion VMT. Estimated on N = 951 state-years (full sample) and N = 931 (excluding Florida). DID: difference-in-differences; VMT: vehicle miles traveled.

Sample	τ₀	τ₁	τ₂	τ₃	τ₄
Full sample	0.041 (0.018)	0.053 (0.021)	0.045 (0.023)	0.065 (0.024)	0.060 (0.027)
Excluding Florida	0.042 (0.018)	0.055 (0.021)	0.046 (0.023)	0.066 (0.024)	0.060 (0.027)

Appendix H

**Table 10:** Sensitivity analysis excluding Florida — dynamic treatment effects (τ₀–τ₄), retail timing Note. Cell entries are event-time coefficients with standard errors in parentheses. Event time 0 is the year retail sales began. Estimates from the Borusyak et al. [33] imputation-based DID estimator (did_imputation) with state and year fixed effects, time-varying covariates as in the primary specification, and standard errors clustered by state. Coefficients are in log points of the traffic fatality rate per 1 billion VMT. Estimated on N = 971 state-years (full sample) and N = 951 (excluding Florida). DID: difference-in-differences; VMT: vehicle miles traveled.

Sample	τ₀	τ₁	τ₂	τ₃	τ₄
Full sample	0.011 (0.020)	0.037 (0.020)	0.015 (0.020)	0.044 (0.034)	0.047 (0.020)
Excluding Florida	0.012 (0.020)	0.037 (0.020)	0.015 (0.021)	0.045 (0.034)	0.048 (0.020)

Appendix I

**Table 11:** Imputation-based difference-in-differences event-study estimates for log traffic fatality rate per 1 billion vehicle miles traveled, with treatment timing indexed to the legalization effective year Note. Estimates are ATT at event time τ relative to the year the state's recreational cannabis law came into effect (τ = 0). This specification differs from the primary analysis (which indexes τ to the first calendar year of legalization) by using the statutory effective date, accommodating the lag between passage and implementation. The outcome is the natural log of the fatality rate per 1 billion vehicle miles traveled. The model includes state and year fixed effects and the same time-varying covariates as the primary specification (Table 2). Percentage change is calculated as (exp(coefficient) − 1) × 100. Standard errors are clustered by state. The joint pre-trend test evaluates the null hypothesis that all pre-treatment leads (τ = −4 through τ = −1) are jointly zero; the joint post-treatment test evaluates the null that all post-treatment lags (τ = 0 through τ = 4) are jointly zero. The results are qualitatively identical to the primary passage-year specification, supporting robustness of the headline finding to the definition of legalization timing. ATT: average treatment effect on the treated; VMT: vehicle miles traveled.

Event time (years)	Coefficient	SE	95% CI	p	% Change
−4	0.018	0.021	[−0.022, 0.059]	.368	+1.9%
−3	0.025	0.016	[−0.008, 0.057]	.134	+2.5%
−2	0.023	0.025	[−0.026, 0.072]	.351	+2.4%
−1	0.029	0.021	[−0.012, 0.071]	.160	+3.0%
0 (effective legalization year)	0.045	0.018	[0.010, 0.081]	.013	+4.6%
1	0.072	0.025	[0.024, 0.120]	.003	+7.4%
2	0.030	0.018	[−0.004, 0.065]	.084	+3.1%
3	0.062	0.020	[0.023, 0.102]	.002	+6.4%
4	0.039	0.029	[−0.018, 0.096]	.180	+4.0%
Joint pre-trend test	χ²(4) = 3.74		—	.443	—
Joint post-treatment test	χ²(5) = 12.59		—	.028	—
State-years (N)	957				

Appendix J 

**Table 12:** Poisson event-study estimates for traffic fatality counts with vehicle miles traveled as an exposure offset Estimates are coefficients from a Poisson event-study model of annual traffic fatality counts with the natural log of vehicle miles traveled included as an exposure offset and include state and year fixed effects. The model includes state and year fixed effects and adjusts for the same time-varying covariates as the primary specification. Event time is defined relative to the year recreational cannabis legalization took effect (τ = 0); τ = −1 serves as the reference period and is omitted from estimation. Standard errors are clustered by state. IRR values are calculated as exp(coefficient). The joint pre-trend test evaluates the null hypothesis that all estimated pre-treatment leads (τ = −4, −3, and −2) are jointly zero; the joint post-treatment test evaluates the null that all post-treatment lags (τ = 0 through τ = 4) are jointly zero. IRR: incidence rate ratio; SE: standard error; VMT: vehicle miles traveled

Event time (years)	Coefficient	SE	95% CI	P-value	IRR
−4	−0.011	0.017	−0.044, 0.022	0.523	0.989
−3	0.027	0.016	−0.005, 0.058	0.097	1.027
−2	0.003	0.020	−0.036, 0.042	0.880	1.003
−1 (reference)	—	—	—	—	1.000
0 (legalization year)	0.040	0.019	0.003, 0.076	0.033	1.040
1	0.062	0.024	0.015, 0.109	0.010	1.064
2	0.034	0.036	−0.037, 0.105	0.346	1.035
3	0.074	0.024	0.027, 0.121	0.002	1.077
4	0.120	0.032	0.059, 0.182	<0.001	1.128
Joint pre-trend test	χ²(3) = 6.42	—	—	0.093	—
Joint post-treatment test	χ²(5) = 17.86	—	—	0.003	—
State-years (N)	951				
